# Effect of thalamic deep brain stimulation on swallowing in patients with essential tremor

**DOI:** 10.1002/acn3.51099

**Published:** 2020-06-16

**Authors:** Sriramya Lapa, Inga Claus, Sarah C. Reitz, Johanna Quick‐Weller, Sonja Sauer, Sigrid Colbow, Christiane Nasari, Rainer Dziewas, Jun‐Suk Kang, Simon Baudrexel, Tobias Warnecke

**Affiliations:** ^1^ Department of Neurology University Hospital Frankfurt Frankfurt Germany; ^2^ Department of Neurology University Hospital Muenster Muenster Germany; ^3^ Department of Neurosurgery University Hospital Frankfurt Frankfurt Germany

## Abstract

**Objective:**

Deep brain stimulation (DBS) of the ventral intermediate nucleus (VIM) is a mainstay treatment for severe and drug‐refractory essential tremor (ET). Although stimulation‐induced dysarthria has been extensively described, possible impairment of swallowing has not been systematically investigated yet.

**Methods:**

Twelve patients with ET and bilateral VIM‐DBS with self‐reported dysphagia after VIM‐DBS were included. Swallowing function was assessed clinically and using by flexible endoscopic evaluation of swallowing in the stim‐ON and in the stim‐OFF condition. Presence, severity, and improvement of dysphagia were recorded.

**Results:**

During stim‐ON, the presence of dysphagia could be objectified in all patients, 42% showing mild, 42% moderate, and 16 % severe dysphagia. During stim‐OFF, all patients experienced a statistically significant improvement of swallowing function.

**Interpretation:**

VIM‐DBS may have an impact on swallowing physiology in ET‐patients. Further studies to elucidate the prevalence and underlying pathophysiological mechanisms are warranted.

## Introduction

Essential tremor (ET) is the most common movement disorder with a prevalence of 0.9%.^1^, ^2^ Thalamic deep brain stimulation (DBS) is a mainstay of treatment for severe and drug‐refractory ET.^3^, ^4^ However, postoperative management may be challenging.^5^ As the most frequent side effect, stimulation‐induced dysarthria (SID) has been reported with an average occurrence of 9%, with values ranging up to 75%.^6^

The exact pathogenesis remains unknown, but it is hypothesized that current spread affecting neighboring structures causes SID. This could be either due to interference with physiological cerebellar information, or due to affection of corticobulbar fiber tracts of the internal capsule.^7^, ^8^, ^9^, ^10^ In addition, both cerebellum and corticobulbar fibers play an important role in the process of swallowing with the latter carrying information from the motor cortex to the cranial nerve nuclei innervating the swallowing musculature and the former being responsible for coordination, sequencing, and timing of swallowing function.^11^, ^12^, ^13^, ^14^, ^15^ Considering this substantial neuroanatomical overlap of structures involved in control and execution of speech and swallowing, it can be assumed that both stimulation of the internal capsule or interference with the cerebellar network might affect swallowing physiology resulting in stimulation‐induced dysphagia. However, in contrast to SID, possible impairment of swallowing function after DBS of the ventral intermedius nuclei (VIM‐DBS) has not been systematically investigated yet. The aim of this study was to evaluate the impact of VIM‐DBS on swallowing function in patients with self‐reported dysphagia using flexible endoscopic evaluation of swallowing (FEES). In addition, information about the underlying pathology was obtained by analyzing the observed pattern of dysphagia.

## Methods

We retrospectively evaluated patients with ET and VIM‐DBS who had received standardized FEES in the DBS‐ON and OFF condition for swallowing assessment at the dysphagia outpatient clinics of the German University Hospitals of Frankfurt and Muenster between 2011 and 2017. In total, 12 patients were included. All subjects reported swallowing problems which had developed during the course of DBS treatment. Detailed medical history was obtained from every subject and there was no evidence of any other diagnosis as the underlying cause of dysphagia. Clinical examination was performed by a neurologist; additional assessment of speech and swallowing function by a speech and language pathologist (SLP).

All patients received standardized FEES in the DBS‐ON condition and after deactivation of the stimulator for a variable time.^16^, ^17^ Oropharyngeal dysphagia was deemed to be present when one or more pathological findings (e.g., penetration/aspiration, residue) occurred during FEES.^18^ The study was approved by the ethics committees of the Goethe University Hospital Frankfurt and University Hospital of Muenster and was conducted according to the principles of the Declaration of Helsinki.

### Stim‐ON and Stim‐OFF assessment of swallowing function

FEES was performed by a neurologist and a SLP in Muenster and two SLPs in Frankfurt. FEES equipment consisted of a 3.1‐mm‐diameter flexible fiberoptic rhinolaryngoscope (ENF‐P4, Olympus, Hamburg, Germany), light source (Storz, Endovision Telecam, SL pal 20212020, Storz, Tuttlingen, Germany), camera (Storz, Endovision Telecam, SLpal 20212030), color monitor (Sony, DVM 14M2MDE, Tokyo, Japan), and videorecorder (Sony, SVO9500MDP) (Muenster) and of a 3.1‐mm‐diameter flexible fiberoptic rhinolaryngoscope (ENF‐P4, Olympus), 150W light source for endoscopic application (rp‐150), camera (rpCam62, S/N), color monitor (7′‐TFT‐EIZO, 1500:1), and a videorecorder (1/2” CCD‐Kamera, rp Cam62, Sony) (Frankfurt).

Patients were assessed at two different time points in the following conditions: (1) during stim‐ON with clinically optimized and chronically used stimulation parameters and (2) during stim‐OFF after the DBS has been deactivated for variable time interval (range 1–96 h).

We followed a standardized FEES protocol as published before.^18^. FEES videos were rated according to a standardized dysphagia score which had been developed for assessing treatment effects on swallowing function in patients with movement disorders.^19^, ^20^, ^21^, ^22^ In brief, three salient parameters of swallowing function were evaluated and scored: (I) premature spillage, (II) penetration‐aspiration events, (III) residue. Premature spillage was defined as when the bolus spilled into the pharynx prior volitional posterior lingual propulsion and was distinguished from delayed swallow by identifying purposeful transfer of the bolus into the pharynx.^23^ Scores of all single ratings were added yielding a total dysphagia sum score with a range from 0 to 108 and higher scores indicating worse function.

FEES examinations were video‐recorded and stored on a hard disc for later review (Muenster) or saved on an external server (Frankfurt). All videos, that is, stim‐OFF and stim‐ON FEES assessments, were independently scored by two raters who were blinded for the patients' clinical data and assessment conditions. For final analysis of the results disagreements were discussed until agreement was reached. Severity of swallowing dysfunction was classified according to a previously published scale which ranges from 0 (no dysphagia) to 3 (severe dysphagia).^22^

### Statistical analysis

Statistical analyses were performed with R (version 3.4.4) and SPSS 19 (IBM Corporation, Somers, NY). Dysphagia sum scores were compared between the stim‐ON and stim‐OFF condition using the Wilcoxon signed rank test. Interrater reliability was analyzed separately for every single FEES dysphagia subscore (premature spillage, penetration‐aspiration events, residue) for both conditions using ranked correlation (ICC by Friedmann chi‐square procedure) providing a Cronbach's alpha coefficient. Spearman's rank correlation coefficient was used to analyze the correlation between dysphagia severity and the total electrical energy delivered (TEED) by DBS, for all patients for which the respective data were available. All tests were performed two‐sided and considered significant when *P*‐values were <0.05.

## Results

Twelve patients (4 female) were included the study (Frankfurt *n* = 8; Muenster *n* = 4). All patients suffered from action and postural tremor, whereas resting tremor was present in 4, intention tremor in 10, and head tremor in three patients.

Average age was 69 ± 9 years; disease duration 33 ± 21 years and time from electrode implantation to dysphagia assessment was 26 ± 24 months. Time between DBS and onset of subjective dysphagia was 12 ± 10 months. Other reported side effects were dysarthria (7/12; 58%), gait ataxia (4/12; 33%), and limb ataxia (2/12; 17%). All patients had a marked tremor reduction during stim‐ON with significant improvement in hand function, handwriting, and activities of daily routine which was both self‐reported by the patient and also observed by the neurologist and SLP. The average time between DBS deactivation and stim‐OFF FEES amounted to 33 ± 28 h (range 1–96 h) (Table 1). Neurological examination revealed no evidence of concomitant diseases causing dysphagia.

**Table 1:** Demographic and clinical data of the patients.

*N*	Gender/age	IPG	Stimulation parameters	FEES dysphagia score ON	FEES dysphagia score OFF	Improvement %	Stimulation off (h)
			Right contact					Right (mA)	Right (µsec)	Right (Hz)	Left contact	Left (mA)	Left (µsec)	Left (Hz)
1	m/78	Medtronic Kinetra	8−/9+	5.6	120	130	0−/G+	3.8	90	130	28	8	71.4	48
2	m/45	Medtronic Activa PC	9−/G+	3	60	150	0−/G+	2.7	60	150	15	2	84.7	48
3	m/70	Medtronic Kinetra	Off	–	–	–	1−/G+	3.8	60	120	19	3	84.2	48
4	f/61	Medtronic Kinetra	8−/9+	1.1	210	130	0−/1+	2.2	210	130	12	2	83.3	24
5	f/70	Medtronic Activa PC	9−/G+	2.3	90	150	1−/G+	4.1	90	150	42	4	90.4	96
6	f/68	Medtronic Activa PC	9−/G+	3.8	90	130	2−/G+	2.3	90	130	20	6	70	48
7	m/80	Medtronic Activa PC	9−/G+	3.9	60	160	0−/G+	3.4	60	160	13	4	69.2	48
8	f/67	Medtronic Activa PC	9−/G+	2.7	60	130	0−/G+	2.1	60	130	5	0	100	17
9	m/70	Medtronic Activa PC	1−/G+	1.7	60	130	9−/G+	2.6	60	130	6	1	83.3	12
10	m/79	Medtronic Activa PC	1−/2+	3.1	60	220	8−/11+	4.2	60	220	17	5	70.6	2
11	m/71	Medtronic Activa PC	9−/10+	n/a	60	130	1−/2+	n/a	60	130	14	3	78.6	1
12	m/66	Boston Vercise	1−(14%)/ 2−(65%)/ 3−(21%)/G+	3.4	60	130	2−(100%)/ G+	2.9	60	130	7	0	100	1

FEES, fiberoptic evaluation of swallowing; IPG, implantable pulse generator; n/a, not available.

Dysphagia was present in all patients in the stim‐ON condition (*n* = 12), the average FEES dysphagia sum score amounting to 16 ± 10 (range 5–42). The most common FEES findings during stim‐ON were premature spillage of the entire bolus and/or of bolus parts with the consequence of quick and uncontrolled overflow into laryngeal vestibule in 83% (10/12) as well as predeglutitive penetration in 58% (7/12) and predeglutitive aspiration in 25% (3/12) of cases (Table 2). 71% (5/7) of the penetration and all the aspiration (3/3) events were directly related to the premature spillage. Of note, swallowing impairment was observed testing all consistencies (11/12 liquid, 10/12 semisolid, 9/12 solid) with premature spillage occurring mostly during swallowing of liquid textures. 50% of the patients (6/12) showed oral residues with fragmented bolus transfer. Pharyngeal residues were observed in about 60% of subjects which were primarily present when semisolid (7/12) and/or solid food (8/12) were applied. Dysphagia was classified as mild in 42% (5/12) and as moderate in 42% (5/12) of patients, whereas 16% (2/12) of patients suffered from severe dysphagia. In the stim‐OFF condition, the mean FEES dysphagia sum score decreased to 3 ± 2 which translates to an average improvement of 82% compared to stim‐ON (*P* = 0.003, Wilcoxon signed‐rank test, sum of signed ranks = 78) (Table 3). Dysphagia severity was classified as mild in 3/12, moderate in 1/12, and severe in 1/12 patient, whereas in 7/12 subjects swallowing function was evaluated as normal.

**Table 2:** Individual DBS parameters of the patients receiving reprogramming.

Subjects	Stimulation parameters chronic use	Stimulation parameters after re‐programming
*N*	Gender/Age	IPG	Right contact	Right (mA)	Right (µsec)	Right (Hz)	Left contact	Left (mA)	Left (µsec)	Left (Hz)	Right contact	Right (mA)	Right (µsec)	Right (Hz)	Left contact	Left (mA)	Left (µsec)	Left (Hz)
1	m/78	Medtronic Kinetra	8−/9+	5.6	120	130	0−/G+	3.8	90	130	8−/9+	3.1	120	130	0−/G+	2.6	90	130
4	f/61	Medtronic Kinetra	8−/9+	1.1	210	130	0−/1+	2.2	210	130	8−/9+	1	120	130	0−/1+	1.9	120	130
5	f/70	Medtronic Activa PC	9−/G+	2.3	90	150	1−/G+	4.1	90	150	9−/10+	2.0	60	130	0−/1+	4.2	60	130
6	f/68	Medtronic Activa PC	9−/G+	3.8	90	130	2−/G+	2.3	90	130	9−/G+	2.3	90	130	2−/G+	2.3	90	130
9	m/70	Medtronic Activa PC	1−/G+	1.7	60	130	9−/G+	2.6	60	130	1−/G+	3.1	60	130	9−/10+	2.5	60	130
10	m/79	Medtronic Activa PC	1−/2+	3.2	60	220	8−/11+	4.2	60	220	2−/3+	3.4	60	180	10−/11+	3.8	60	180

DBS, deep brain stimulation.

**Table 3:** Individual FEES dysphagia scores.

*N*	Gender/age	FEES parameters stim‐ON	FEES parameters stim‐OFF
		Leaking	Penetration/aspiration	Residue	Sum score	Leaking	Penetration/aspiration	Residue	Sum score
1	m/78	12	16	0	28	2	3	3	8
2	m/45	8	1	6	15	1	0	1	2
3	m/70	13	0	6	19	3	0	0	3
4	f/61	4	0	8	12	2	0	0	2
5	f/70	12	13	17	42	1	2	1	4
6	f/68	11	5	4	20	6	0	0	6
7	m/80	4	0	9	13	0	0	4	4
8	f/67	2	0	3	5	0	0	0	0
9	m/70	6	0	0	6	1	0	0	1
10	m/79	14	0	3	17	3	0	2	5
11	m/71	14	0	0	14	3	0	0	3
12	m/66	7	0	0	7	0	0	0	0

FEES, fiberoptic endoscopic evaluation of swallowing.

Noteworthy, swallowing completely recovered in two patients, whereas in the remaining 10, subtle pathological findings maintained (range 1–8 points) Demographic and clinical data are presented in Table 1.

Correlation of dysphagia severity and TEED (averaged over both sides) was statistically significant (*r* = 0.71, *P* = 0.028, Spearman's rank correlation coefficient).

Interclass correlation analyses demonstrated very good interrater reliability for all single parameters (premature spillage, penetration‐aspiration events, residue) and both DBS conditions with Cronbach's alpha ranging from 0.84 to 0.90 (stim‐ON) and from 0.82 to 0.95 (stim‐OFF).

## Discussion

In this study, we evaluated the impact of VIM‐DBS on swallowing function in a sample of ET patients suffering from dysphagia. Although dysarthria is a well‐known side effect of VIM‐DBS, this is – to the best of our knowledge – the first systematic and instrumental‐based report on dysphagia as a VIM‐DBS‐induced adverse effect. In all investigated cases, dysphagia was confirmed using FEES when DBS was on. After DBS deactivation dysphagia significantly improved in all patients, the mean improvement of FEES dysphagia sum score amounting to 80%. Reason for lack of full recovery could be age‐ or disease‐related changes in swallowing which we cannot completely rule out because no FEES assessment was done before surgery.^24^ Likewise, the deactivation period of the neuro‐stimulator may not have been long enough to completely resolve swallowing function.^1^ At present, the amount of time needed for the DBS to be turned off in order to allow for a noticeable change in the patient's swallow remains elusive and should be investigated in future studies.

In analogy to SID, two pathophysiologic mechanisms can be hypothesized that may underlie dysphagia in VIM‐DBS, namely: (1) via an unintended stimulation of corticobulbar fibers in the internal capsule^9^ or (2) via DBS‐induced modulation of the cerebellar network due to stimulation of cerebellar‐thalamic afferents.^25^ The analysis of the dysphagia pattern rather supports the latter for the following reasons: The main endoscopic finding was premature spillage with quick overflow into the laryngeal vestibule accompanied with penetration/aspiration before swallowing. Thus, bolus control and transition from oral to pharyngeal stage are affected by a lack of coordination of the muscles of the oral cavity rather than delayed pharyngeal response. This observation is more likely caused by interference of DBS with cerebellar circuits resulting in an ataxic dysphagia pattern.^26^, ^27^, ^28^

This view is supported by the fact that all patients reported a considerable delay between the beginning of DBS treatment and the onset of dysphagia. A similar delay in onset was also observed for progressive gait ataxia as a side effect of VIM‐DBS for the treatment of ET.^1^, ^29^ In affected patients, gait ataxia improved within several days after DBS deactivation. The side effect was interpreted as a maladaptive response of distinct cerebellar subregions caused by antidromic stimulation of cerebello‐thalamic afferents in the subthalamic area.^1^, ^29^

Furthermore, it is well‐known from lesion studies that patients suffering from stroke in the internal capsule typically show longer pharyngeal transit times with delayed triggering of pharyngeal swallow,^30^ a pattern which was not observed in our DBS cohort. Noteworthy, all our patients were highly aware of their swallowing difficulties although dysphagia was only mild to moderate in most cases. Patients were not suffering from sensory loss, so cough and/or sustained swallowing were observed as frequent response to penetration/aspiration and/or residue. If stimulation of the internal capsule was the underlying cause of these dysphagic symptoms, additional pharyngolaryngeal sensory deficits should have been observed.^31^, ^32^

Taken together, our clinical findings support the hypothesis that dysphagia more likely results from modulation of cerebellar circuits rather than from direct stimulation of corticobulbar fibers in the internal capsule. However, this hypothesis has to be tested in future studies, for example, using diffusion tensor imaging and tractography in order to assess the overlap of the stimulation field with crucial fiber tracts.

Of note, clinically examination revealed no signs of dysarthria in 42% of the patients. This clearly argues for dysphagia being an independent side effect of DBS treatment.

In general, dysphagia was mild to moderate but nevertheless impacted the patients' well‐being. In six patients, adjustment of the stimulation settings led to full recovery of swallowing function (Table 2). If readjustment of stimulation parameters did not result in a marked and lasting improvement of swallowing, dysphagic symptoms had to be tolerated for the sake of sufficient tremor control.

Additionally, we detected a significant correlation of dysphagia severity and TEED. While our findings are suggestive that patients with high stimulation settings may have a higher risk for developing dysphagia, – due to the low number of cases – this observation must be validated in future studies.

Limitations of this study include its retrospective design and the small sample size thus limiting statistical power. Furthermore, our hypothesis of a cerebellar pathomechanism underlying stimulation‐induced dysphagia is based on clinical observations and, thus, speculative. Future studies are needed which address this issue by analyzing the anatomical correlations between lead locations and the stimulation field with critical areas and fiber tracts.

Nevertheless, this is the first data showing that dysphagia can be a clinically relevant adverse event of VIM‐DBS in ET that should arouse special awareness of the multidisciplinary team in charge of the patient.

Professional assessment of swallowing impairment should be routinely implemented in patients with ET before and after surgery. For quantification of dysphagia, the applied FEES dysphagia score is a suitable tool to evaluate treatment induced improvement or worsening of swallowing function in patients with movement disorders and tremor beyond Parkinson's disease.

Prospective, controlled studies are warranted to gather robust data on the incidence and underlying pathomechanism causing swallowing disorders in VIM‐DBS ET patients in order to optimize patient management.

## Authors' Contributions

Sriramya Lapa, Jun‐Suk Kang, Simon Baudrexel, and Tobias Warnecke involved in drafting the article or revising it critically for important intellectual content. All authors involved in conception and design of the study, or acquisition of data, or analysis and interpretation of data and approved the final submitted version.

## Conflict(s) of Interest and Disclosure(s)

For this study or in the context of this study, all authors have not received any funding from any institution, including personal relationships, interest, grants, employment, affiliations, patents, inventions, honoraria, and consultancies.

Outside the submitted work, Dr. Kang received honoraria from Medtronic, Boston Scientific, Merz Pharmaceuticals, Desitin. Prof. Warnecke received lecture fees from Bial, Abbvie, Licher, Desitin and funding from Abbvie for the Parkinson´s Network Muensterland+(PNM+). Additionally, he is a consultant for Phagenesis. All other authors declare that they have no conflict of interest.
